# Accelerometry-assessed physical activity and sedentary time and associations with chronic disease and hospital visits - a prospective cohort study with 15 years follow-up

**DOI:** 10.1186/s12966-019-0878-2

**Published:** 2019-12-09

**Authors:** Ing-Mari Dohrn, Anna-Karin Welmer, Maria Hagströmer

**Affiliations:** 1 Department of Neurobiology, Care Sciences and Society (NVS), Karolinska Institutet, Aging Research Center, Tomtebodavägen 18A, SE-171 65 Solna, Sweden; 2 Functional Area Occupational Therapy and Physiotherapy, Karolinska University Hospital, SE-171 76 Solna, Sweden; 3 Department of Neurobiology, Care Sciences and Society (NVS), Karolinska Institutet, Division of Physiotherapy, Alfred Nobels allé 23, SE-141 52 Huddinge, Sweden; 4 Department of Health Promoting Science, Sophiahemmet University, Valhallavägen 91, SE-114 86 Stockholm, Sweden

**Keywords:** Accelerometer, Cardiovascular disease, Chronic disease, Hospital admission, Moderate-to-vigorous physical activity, Morbidity, Objective assessment, Population-based, Sedentary behavior

## Abstract

**Background:**

Associations of objectively assessed physical activity in different intensities and risk of developing chronic disease that requires hospital care have not yet been examined in long term population-based studies. Studies addressing the link between physical activity and sedentary time and subsequent hospital admissions are lacking.

**Objective:**

To examine the prospective associations between physical activity and sedentary time with morbidity defined as: 1) a registered main diagnosis of cardiovascular disease, cancer, type-2 diabetes, dementia, obesity or depression; 2) number of in- and outpatient hospital visits; and 3) number of in-hospital days.

**Methods:**

In total, 1220 women and men, 18–75 years, from the population-based Sweden Attitude Behaviour and Change study 2000–2001 were included. Time spent sedentary, in light-intensity physical activity and in moderate-to-vigorous physical activity, and total accelerometer counts were assessed using the ActiGraph 7164 accelerometer. Morbidity data were obtained 2016 from Swedish registers. Cox proportional hazards models estimated hazard ratios (HR) of morbidity with 95% confidence intervals (CI) and negative binomial regression estimated incidence rate ratio (IRR) with 95% CI for number of hospital visits, and length of hospital stay.

**Results:**

Over a follow-up of 14.4 years (SD = 1.6), 342 persons had at least one registered hospital visit due to any of the included diagnoses. Higher moderate-to-vigorous physical activity was associated with significant risk reductions for combined morbidity (all included diagnoses) (HR: 0.65, 95% CI: 0.48–0.88) and cardiovascular disease (HR: 0.52, 95% CI: 0.33–0.82). Higher total counts showed similar results, and was also associated with fewer hospital visits (IRR = 0.56, 95% CI: 0.37–0.85). Higher sedentary time increased the risk of in-hospital days. (IRR = 2.38, 95% CI: 1.20–4.74).

**Conclusion:**

This study supports the importance of moderate-to-vigorous physical activity for preventing chronic disease that requires hospital care, especially cardiovascular disease. High volumes of sedentary behavior may increase the risk of future hospitalization. Our results support the public health message “sit less and move more”.

## Introduction

The benefits of physical activity (PA) are well established and include a reduced risk of many of the most common chronic diseases, such as cardio-vascular disease (CVD), cancer, type-2 diabetes, dementia, and depression [1], Additionally, increasing evidence suggests that high levels of sedentary time may increase the risk of CVD, type-2 diabetes and obesity [1–3], and that people with low levels of PA use significantly more healthcare services than active people [4–6].

However, the current evidence on the associations between PA or sedentary time and morbidity are mainly based on self-reported PA data, which are prone to reporting bias and have limited ability to identify light-intensity PA (activities of everyday living, such as household chores) and sedentary behavior [2, 7, 8]. With recent advancements in movement sensor technologies, portable devices such as accelerometers, have become available in PA research, allowing objective assessments of PA [9, 10]. These devices provide a more accurate investigation of PA through the whole intensity spectra, including light-intensity PA and sedentary time. Due to this relatively new technology, the prospective studies that have examined the associations of objectively assessed PA in different intensities and the risk of developing chronic disease are few and have mainly focused on CVD [11–14], and no previous population-based study has a follow-up time as long as 15 years. Further, studies addressing the link between PA and sedentary time and subsequent hospital admissions are lacking. By using accelerometry we can get more accurate knowledge of the risk of morbidity in common diseases previously found to be related to self-reported PA or sedentary behavior. This could guide the design of effective health promotion efforts that may contribute to more healthy years for individuals and save societal costs through reduced use of hospital care.

In this study we used a nationally representative sample of adult women and men to investigate the associations of accelerometer assessed PA and sedentary time and morbidity during a 15-year follow-up period. Our specific aims were to examine the prospective associations between PA and sedentary time with morbidity defined as: 1) a registered main diagnosis of CVD, cancer, type-2 diabetes, dementia, obesity or depression; 2) number of in- and outpatient hospital visits; and 3) number of in-hospital days.

## Material and methods

### Study population

This prospective cohort study used data from the Sweden Attitude Behaviour and Change (ABC) study collected from September 2000 to December 2001 [15, 16]. In the ABC study a random sample of 3300 adults aged 18–75 years (52% women) were selected from the Swedish population register, 2262 were reached by phone and invited to participate and 1556 persons (69%) accepted to participate. In this study, we included the 1220 participants who provided valid physical activity data. This final sample was evenly distributed across Sweden, although the proportion of women were slightly higher, and proportions of participants under 24 years and over 65 years were slightly lower than in the general population [16].

### Baseline data collection

Physical activity was assessed with the ActiGraph 7164 (Pensacola, FL, USA) accelerometer, a small, lightweight device measuring time-varying acceleration in the vertical axis recorded as activity “counts” that can be translated to intensity and duration of PA. The accelerometers, attached to an elastic belt, were delivered and returned by post together with a baseline questionnaire. Participants were instructed to wear the accelerometer on the lower back for seven consecutive days during waking hours, except during water-based activities. Days with > 10 h of accelerometer wear time were considered as valid and participants providing at least one valid day were included [7, 17]. Non-wear time was defined as an interval of at least 60 consecutive minutes of zero counts, with allowance for up to 2 min of 1–100 counts [18]. Cutoff points for PA intensities were < 100 cpm for sedentary time, 100–2019 cpm for light-intensity PA, and ≥ 2020 cpm for moderate-to-vigorous PA (MVPA) [7, 19]. Tertiles of time (in min) spent sedentary, in light-intensity PA, and in MVPA, and tertiles of total activity counts [20] (reflecting total PA) were used as exposure variables.

The questionnaire provided information on age, sex, smoking status (never/former or current), length and weight, history of hypertension, heart disease, cancer, diabetes, or arthritis (yes/no), and education (less than high school, high school/equivalent diploma, or university degree). The ABC study has been described in detail elsewhere [15, 16].

### Follow-up data collection

Register data of morbidity 2002–2015 were obtained in 2016 from the National Patient Register in Sweden. The register includes all inpatient care in Sweden and also covers hospital outpatient visits including day surgery and psychiatric care from both private and public caregivers. (Primary health care visits are not included.) Information on all visits registered with the following six diagnoses, registered as main diagnosis, according to the International Classification of Disease (ICD-10) were retrieved: CVD including stroke, (I10-I15, I20-I25, I60-I79), cancer (C00-D48), diabetes (E10-E14), obesity (E65-E68), dementia (F00, F01, F03) and depression (F32-F39). Benign tumors, subarachnoid hemorrhage, type-1 diabetes, and essential hypertension were excluded. Dates of admission and discharge were used for inpatient care and date of visit for outpatient care. Additional information on cancer diagnoses 2002–2014 was obtained from the Swedish Cancer Register. Information of deaths 2002–2015 was obtained from the Swedish Cause of Death Register for censoring purposes.

### Data analyses and statistics

Time from baseline to the first registered main diagnosis of CVD, cancer, type-2 diabetes, dementia, obesity and depression respectively, were used as primary outcomes in separate time-to-event analyses. Follow-up extended from the first day of accelerometer assessment until the date of death or censoring on December 31, 2015. Individuals that did not experience any of the included diseases were censored at their date of death or at end of follow-up.

Kaplan-Meier survival curves were calculated separately for all six diagnoses and for combined morbidity (i.e. events from any of all six diagnoses). Cox proportional-hazard models were applied to estimate hazard ratios (HR) with 95% confidence intervals (CI) of combined morbidity, excluding participants reporting heart disease, cancer, diabetes or with missing disease history at baseline. The outcome was defined as time from baseline to the date when the first of the above-mentioned diseases occurred, or censoring. Only two diagnoses, CVD and cancer, were analyzed separately with the Cox model, since the other diagnoses were too rare in the study population to provide enough power for the statistical analysis. For the CVD and cancer outcomes, participants with reported heart disease and cancer at baseline were excluded in the respective analysis. Time to first event was calculated for each diagnosis separately, e.g. an individual censored for a CVD event was still included in the cancer analyses. We examined a-priori selected covariates for confounding based on previous literature [21–23], and after assessment of the proportional-hazards assumption, the final adjusted models included age, sex, education, smoking (model 2) and additionally (model 3), diabetes, arthritis and hypertension at baseline. All models for sedentary time were also adjusted for wear time. Participants with missing data for covariates (smoking, *n* = 5, education, *n* = 4, history of disease *n* = 13) were excluded in the adjusted models. Sensitivity analyses were computed to assess the association between the exposure variables and the outcome, using penalized spline functions, but the conclusions did not deviate from our main models. Additional sensitivity analyses were computed for: combined morbidity including only three diagnoses, i.e. CVD, cancer and type-2 diabetes; for CVD incidence excluding stroke; and with BMI as an additional confounder. To limit the possibility of reversed causality, sensitivity analyses were also computed for all Cox proportional-hazard models, excluding participants with events registered during the first three years of follow-up.

Secondary outcomes of interest were number of hospital visits and number of in-hospital days. Number of in- and outpatient visits were merged into one variable, hospital visits. Number of in-hospital days was calculated from the day of admission to and including the day of discharge. If admission and discharge were registered on the same day, one day was included. Negative binomial regression was used to estimate incidence rate ratio (IRR) with 95% CI for number of hospital visits and number of in-hospital days for combined morbidity [24], including the same covariates as in the Cox proportional-hazard models.

Differences between participants without and with registered diagnoses were examined using Student’s t-test or chi-2 test for background characteristics. Level of significance was set at *p* < 0.05 for all analyses. The statistical analyses were computed using the R software version 3.4.2 and STATA 15 (StataCorp, College Station, Texas).

## Results

We followed 1220 adults over a mean of 14.4 years (SD = 1.6). During follow-up 342 persons (28%) had at least one diagnosis registered and 80 deaths (6.5%) occurred. Characteristics of the study population are presented in Table 1. Participants with registered diagnoses were older and less educated than those without diagnoses. There were no statistically significant differences in sedentary time or light-intensity PA observed between participants with or without a registered diagnosis when adjusting for wear time, while those with a registered diagnosis had lower MVPA and total activity counts per day. The accelerometers were worn 14.4 (±1.3) h per day during 6 (±1) days, and 1168 participants (96%) had at least 4 valid days, with no differences in valid wear time between participants with or without registered diagnoses. On average, 8 h 12 min were spent sedentary, 5 h 40 min in light-intensity PA, and 33 min in MVPA per day for the whole sample. Time spent sedentary, in light-intensity PA and in MVPA, and total activity counts are presented by tertiles in Table 2.

**Table 1:** Baseline characteristics for the whole sample and by group of participants, without and with at least one registered diagnosis of cardiovascular disease, type-2 diabetes, obesity, stroke, cancer, dementia, or depression from an in- or outpatient hospital visit during the 15-year follow-up time

Variables	All	No registered diagnosis	With registered diagnosis
	*n* = 1220	*n* = 878	*n* = 342
Sex, women	666 (55%)	471 (54%)	195 (57%)
Age^a^, years	45.3 (±14.5)	41.5 (±13.3)	54.9 (±12.8)
Smoker^c^	290 (24%)	206 (23%)	84 (25%)
Education^a,c^			
< High school	281 (23%)	150 (17%)	131 (38%)
High school	558 (46%)	442 (51%)	116 (34%)
University degree	377 (31%)	283 (32%)	94 (28%)
History of disease^a,b,c^	211 (17%)	103 (12%)	108 (32%)
Physical activity			
Sedentary time, min/day	491.9 (±91.9)	490.0 (±94.0)	496.8 (±86.3)
Light-intensity PA^a^, min/day	339.5 (±94.9)	343.4 (±95.6)	329.5 (±92.7)
Moderate-to-vigorous PA^a^, min/day	33.5 (±29.5)	35.4 (±26.9)	28.8 (±34.8)
Total activity counts/day^a^ (thousands)	321.0 (±195.9)	331.8 (±162.7)	293.2 (±260.8)

Values presented are mean (±SD) or number (%). ^a^ Difference between participants without and with registered diagnose calculated using Student’s t-test and chi-2 test *p* < 0.05. ^b^ Self-reported current or previous hypertension, cardiovascular disease, diabetes, cancer, arthritis or emphysema at baseline. ^c^ Missing data: smoker, *n* = 5; education, *n* = 4; history of disease *n* = 13

*PA* physical activity

**Table 2:** Median (min-max) time/counts, number of participants and number of events for different PA intensities and total activity counts by tertiles (*n* = 1220)

	Tertile 1	Tertile 2	Tertile 3
Sedentary time, min/day	402 (188–454)	494 (455–529)	578 (530–785)
Participants/events, *n*	407/102	407/119	406/121
Light-intensity PA, min/day	251 (9–295)	337 (296–378)	433 (379–794)
Participants/events, *n*	407/121	407/127	406/64
Moderate-to-vigorous PA, min/day	12 (0–19)	28 (20–38)	54 (39–501)
Participants /events, *n*	411/160	404/103	405/79
Total activity counts/day, thousands	200 (6–250)	300 (251–343)	433 (350–429)
Participants/events, *n*	411/159	405/98	404/85

*PA* physical activity

In total, 451 hospital visits were registered with the following distribution of diagnoses: CVD, *n* = 187; cancer, *n* = 176; type-2 diabetes, *n* = 34; dementia, *n* = 7: obesity, *n* = 16; and depression, *n* = 37. The Kaplan-Meyer analyses showed significant associations between MVPA and combined morbidity, CVD, cancer, obesity and dementia; and between total activity counts and combined morbidity, CVD, cancer, type-2 diabetes, and obesity (Additional file 1: Figure S1).

Table 3 shows HR for crude and adjusted models of combined morbidity by tertiles of sedentary time, light-intensity PA, MVPA, and total activity counts. Table 4 shows HR for crude and adjusted models of CVD and cancer morbidity. Inverse associations with combined morbidity and CVD were observed for MVPA. For cancer morbidity, the crude model showed a significant lower HR for those with the most time in MVPA and with highest number of total activity counts. In the adjusted models these associations were attenuated. Light-intensity PA and sedentary time were not associated with either of combined morbidity, CVD or cancer. None of the sensitivity analyses changed the findings or main conclusions, except that in combined morbidity including only CVD, cancer and type-2 diabetes, the results for MVPA and total counts were attenuated in the adjusted models (Additional file 2: Table S1).

**Table 3:** Associations between physical activity and sedentary time and combined morbidity, i.e. registered hospital visits due to any of the included diagnoses (cardiovascular disease, cancer, type 2-diabetes, dementia, obesity or depression), for participants without reported heart disease, cancer or diabetes at baseline

Combined morbidity	Model 1		Model 2		Model 3	
*n* = 1132 / 286 events	HR	95% CI	HR	95% CI	HR	95% CI
Sedentary time						
Tertile 1	1		1		1	
Tertile 2	**1.33**	**1.00, 1.76**	1.07	0.81, 1.42	1.07	0.80, 1.42
Tertile 3	1.24	0.91, 1.68	1.19	0.88, 1.62	1.18	0.87, 1.61
Light-intensity PA						
Tertile 1	1		1		1	
Tertile 2	1.11	0.83, 1.47	1.07	0.80, 1.42	1.08	0.81, 1.43
Tertile 3	0.89	0.67, 1.19	0.90	0.67, 1.20	0.90	0.67, 1.21
Moderate-to-vigorous PA						
Tertile 1	1		1		1	
Tertile 2	**0.64**	**0.49, 0.84**	0.90	0.68, 1.19	0.90	0.68, 1.20
Tertile 3	**0.44**	**0.32, 0.59**	**0.64**	**0.48, 0.87**	**0.65**	**0.48, 0.88**
Total activity counts						
Tertile 1	1		1		1	
Tertile 2	0.90	0.68, 1.20	0.94	0.71, 1.25	0.94	0.71, 1.25
Tertile 3	**0.54**	**0.41, 0.73**	**0.64**	**0.48, 0.86**	**0.64**	**0.48, 0.86**

Cox proportional-hazard models were used to estimate hazard ratios (HR) with 95% confidence intervals (CI). Model 1: crude. Model 2: adjusted for age, sex, smoking (missing = 5), and education (missing = 4). Model 3: adjusted for model 2 variables plus hypertension and arthritis (missing = 13) at baseline. All models for sedentary time additionally adjusted for wear time. Statistically significant results shown in bold

*PA* physical activity

**Table 4:** Associations between physical activity and cardiovascular disease and cancer for participants without reported heart disease at baseline and without reported cancer at baseline respectively

Cardiovascular disease	Model 1		Model 2		Model 3		Cancer	Model 1		Model 2		Model 3	
*n*= 1,176 / 139 events	HR	95% CI	HR	95% CI	HR	95% CI	*n*=1,181 / 161 events	HR	95% CI	HR	95% CI	HR	95% CI
Sedentary time							Sedentary time						
Tertile 1	1		1		1		Tertile 1	1		1		1	
Tertile 2	1.41	0.91, 2.17	1.06	0.68, 1.65	1.05	0.68, 1.64	Tertile 2	**1.56**	**1.05, 2.30**	1.34	0.90, 1.99	1.35	0.91, 2.00
Tertile 3	**1.85**	**1.20, 2.85**	**1.55**	**1.00, 2.40**	1.41	0.91, 2.20	Tertile 3	1.43	0.95, 2.16	1.37	0.90, 2.07	1.37	0.91, 2.08
Light-intensity PA							Light-intensity PA						
Tertile 1	1		1		1		Tertile 1	1		1		1	
Tertile 2	0.93	0.63, 1.37	0.98	0.66, 1.45	1.03	0.70, 1.53	Tertile 2	1.26	0.88, 1,82	1.18	0.82, 1.70	1.17	0.81, 1.69
Tertile 3	0.67	0.44, 1.03	0.85	0.55, 1.31	0.92	0.60, 1.42	Tertile 3	0.83	0.55, 1,24	0.88	0.58, 1.33	0.87	0.58, 1.32
Moderate-to-vigorous PA							Moderate-to-vigorous PA						
Tertile 1	1		1		1		Tertile 1	1		1		1	
Tertile 2	**0.46**	**0.31, 0.68**	0.74	0.49, 1.12	0.77	0.52, 1.19	Tertile 2	0.72	0.50, 1.04	1.13	0.78, 1.65	1.14	0.78, 1.67
Tertile 3	**0.33**	**0.21, 0.51**	**0.50**	**0.32, 0.78**	**0.52**	**0.33, 0.82**	Tertile 3	**0.60**	**0.41, 0.88**	0.99	0.67, 1.47	0.95	0.67, 1.48
Total activity counts							Total activity counts						
Tertile 1	1		1		1		Tertile 1	1		1		1	
Tertile 2	**0.56**	**0.37, 0.83**	**0.66**	**0.44, 0.99**	0.70	0.46, 1.05	Tertile 2	0.89	0.62, 1.27	1.00	0.69, 1.44	1.00	0.69, 1.44
Tertile 3	**0.50**	**0.23, 0.76**	**0.64**	**0.41, 0.98**	0.67	0.44, 1.04	Tertile 3	**0.66**	**0.44, 0.98**	0.86	0.57, 1.29	0.86	0.57, 1.29

Cox proportional-hazard models were used to estimate hazard ratios (HR) with 95% confidence intervals (CI)

Model 1: crude. Model 2: adjusted for age, sex, smoking (missing=5), and education (missing=4). Model 3: adjusted for model 2 variables plus hypertension, diabetes, and arthritis (missing=13) at baseline. All models for sedentary time additionally adjusted for wear time. Statistically significant results shown in bold

*PA* physical activity

The IRR for hospital visits and in-hospital days from crude and adjusted models are shown in Table 5. Participants with a registered diagnosis had a median of 5 hospital visits, min-max: 1–109. Among the 214 individuals with registered in-hospital visits, median number of visits was 2, min-max: 1–17, and median number of in-hospital days was 10, min-max: 1–189. Higher total activity counts were inversely associated with hospital visits, while high sedentary time showed the strongest associations with more in-hospital days.

**Table 5:** Incidence rate ratio (IRR) with 95% confidence intervals (CI) for number of hospital visits including both in- and out-patient visits (n=1,220, 451 events) and number of in-hospital days

Hospital visits	Model 1		Model 2		Model 3		In-hospital days	Model 1		Model 2		Model 3	
	IRR	95% CI	IRR	95% CI	IRR	95% CI		IRR	95% CI	IRR	95% CI	IRR	95% CI
Sedentary time							Sedentary time						
Tertile 1	1		1		1		Tertile 1	1		1		1	
Tertile 2	1.24	0.79, 1.95	0.77	0.50, 1.19	0.77	0.49, 1.19	Tertile 2	**2.36**	**1.21, 4.63**	**2.51**	**1.34, 4.71**	**2.47**	**1.32, 4.63**
Tertile 3	1.55	0.98, 2.44	1.05	0.67, 1.65	1.05	0.67, 1.64	Tertile 3	**2.38**	**1. 20, 4.74**	**2.14**	**1.11, 4.12**	**2.02**	**1.05, 3.89**
Light-intensity PA							Light-intensity PA						
Tertile 1	1		1		1		Tertile 1	1		1		1	
Tertile 2	0.81	0.52,1.28	0.96	0.62, 1.48	0.98	0.63, 1.52	Tertile 2	0.67	0.34,1.31	0.84	0.45, 1.58	0.87	0.46, 1.63
Tertile 3	**0.58**	**0.37, 0.92**	0.89	0.57, 1.40	0.91	0.58, 1.43	Tertile 3	**0.35**	**0.18, 0.68**	**0.45**	**0.24, 0.86**	**0.49**	**0.26, 0.93**
Moderate-to-vigorous PA							Moderate-to-vigorous PA						
Tertile 1	1		1		1		Tertile 1	1		1		1	
Tertile 2	0.77	0.49, 1.22	1.23	0.78, 1.97	1.24	0.77, 1.98	Tertile 2	0.52	0.27, 1.02	1.11	0.58, 2.13	1.07	0.56, 2.06
Tertile 3	**0.60**	**0.38, 0.94**	1.07	0.68, 1.69	1.07	0.67, 1.71	Tertile 3	**0.44**	**0.22, 0.85**	1.08	0.56, 2.10	1.08	0.56, 2.10
Total activity counts							Total activity counts						
Tertile 1	1		1		1		Tertile 1	1		1		1	
Tertile 2	**0.56**	**0.36, 0.88**	**0.55**	**0.36, 0.84**	**0.56**	**0.37, 0.85**	Tertile 2	**0.44**	**0.23, 0.86**	0.74	0.40, 1.38	0.76	0.41, 1.42
Tertile 3	**0.49**	**0.31, 0.77**	0.78	0.50, 1.20	0.78	0.50, 1.21	Tertile 3	**0.35**	**0.18, 0.69**	0.86	0.45, 1.64	0.89	0.47, 1.69

Model 1: crude. Model 2: adjusted for age, sex, smoking (missing=5), and education (missing=4). Model 3: adjusted for model 2 variables plus hypertension, diabetes, and arthritis (missing=13) at baseline. All sedentary models additionally adjusted for wear time. Statistically significant results shown in bold

*PA* physical activity

## Discussion

The novel aspect of this study was the use of accelerometry to investigate the prospective associations of daily PA and sedentary behavior with risk of chronic disease requiring hospital care in a population-based sample with a follow-up time of 15 years. Our results support what previously has been found in PA research using self-reported data, namely, that many of the most prevalent chronic diseases and most expensive medical conditions are favorably influenced by higher levels of PA, and that the public health burden of sedentary behavior may be substantial [1, 4–6]. The investigated diagnoses are strongly associated with PA and sedentary behavior [1–3], and by collecting data from hospital in- and outpatient care it is reasonable to believe that we included the most serious events, likely to have major consequences for the individual, as well as a high burden for the society. In addition to the reported diagnoses, we used number of hospital visits and number of in-hospital days as measures of morbidity. These outcomes do not only reflect the severity of a condition but also how PA habits contributes to health care costs [1, 4].

We found that MVPA may lower the risks of morbidity, especially CVD morbidity. Individuals in the highest tertile of MVPA, median 54 min per day, had 35% lower risk of being diagnosed with any of the included diseases and 48% lower risk of CVD than those in the lowest tertile, with only 12 min per day (median) in MVPA. This confirms the importance of MVPA found in prospective studies using self-reported PA and in accelerometry studies with shorter follow-up [1, 13, 25]. We found a higher risk of cancer among the most sedentary individuals and lower risk of cancer among those with the most MVPA and total activity counts in the crude models, but these associations were attenuated after adjusting for confounders. Complex associations of PA and cancer have previously been reported in studies of mortality [1, 22], and although the evidence for a causal link is strong for some cancers, the mechanisms by which PA affects the risk of developing cancer differ by cancer site and other influencing factors [26].

Accelerometry allows reliable investigation of associations of morbidity with light-intensity PA which is hard to achieve when self-reports are used. Surprisingly, and in contrast to LaCroix et al. [11], we did not find associations between light-intensity PA and risk of being diagnosed with a chronic disease. Nor did Jefferis et al. [13] in a study on CVD risk, although recent studies have found that if sedentary time is replaced with light-intensity PA, such as everyday activities, the risk of mortality can be reduced [23, 27].

In contrast to the consistent evidence that sedentary behavior is associated with all-cause, CVD and cancer mortality [1, 22, 27, 28] and increases the risk of developing type-2 diabetes [1, 29], we found no associations between sedentary time and being diagnosed with a chronic disease. In this study a relatively low number of individuals were diagnosed type-2 diabetes and we were not able to perform separate calculations. A possible explanation is that patients with type-2 diabetes are mainly treated in primary health care in Sweden, and primary care is not yet covered in the National Patient Register.

Interestingly, even though we did not find that sedentary time was associated with a higher risk of being diagnosed with a chronic disease, we found that the most sedentary individuals had a more than doubled risk of more in-hospital days. Correspondingly, individuals with most light-intensity PA had half the risk compared with those with least light-intensity PA. These are novel findings and it would be interesting to further investigate these associations with use of other healthcare services, such as primary healthcare or medications costs.

Important strengths of this study are the long follow-up time and the highly reliable PA data. We collected data on morbidity from the Swedish National Patient Register, in which the main diagnosis is registered for 99% of all hospital in-patient admissions and 96% of all out-patient visits. The validity is high with 85–95% positive predictive values of ICD codes from medical records [30]. We used device-based assessment of PA and sedentary time with high wear time compliance; a vast majority of our sample had at least four days of recording.

Our study also has several limitations that should be mentioned. The ABC study sample was nationally representative, but as in any study the participants may be healthier and more physically active than the general population. Still, the sample retained a wide range of PA and sedentary behavior levels suggesting that these behaviors were not likely to have introduced bias. Morbidity at baseline was self-reported and thereby less reliable, and information about history of depression and dementia were lacking. Even though the sensitivity analyses excluding diagnoses registered the first three years of follow-up did not change the results, reverse causation is still possible, especially for dementia due to the long preclinical phase [31]. We adjusted for several relevant factors, but as in any observational study, our results may be subject to residual confounding. For example, we did not have information about diet and alcohol consumption, or mobility restrictions.

Despite the accurate information accelerometers can provide about levels and patterns of PA, there are also some methodological limitations: the analyses rely on the chosen cutoff points for classification of intensities [32] and some types of PA cannot be captured, such as upper body movements, biking and swimming [33]. In addition, the ActiGraph records body movement and not postures, and consequently our sedentary time measure may include standing time [34]. As an alternative to intensity classified PA we also used total activity counts per day. Total activity counts provide a measure of accumulated total volume of PA not relying on cutoff points [20]. Total PA contributes to health benefits [1], and recent research using device-measured PA have suggested that total PA may be more important for reducing CVD risks than MVPA [13, 25]. PA was only assessed at baseline and we do not have information about possible changes in PA habits or sedentary behavior during follow-up that may have influenced the observed associations. However, the results from an ABC sub study showed that PA levels were unchanged from 2001 to 2008, suggesting that potential changes are small and will not have a major impact on our results [15].

Finally, it is possible that some results are due to lack of power and more studies with larger sample sizes are needed.

## Conclusion

This study supports the importance of MVPA for preventing chronic disease that requires hospital care, especially CVD. The associations were more complex for cancer. High volumes of sedentary behavior may increase the risk of future hospitalization. Physically active individuals had fewer hospital visits, whereas more sedentary time doubled the risk of spending more days in hospital. Our results support the public health message “sit less and move more”, which is especially important for the least physically active individuals, and have the potential of reducing both individual and societal burden of disease.

## Supplementary information

**Additional file 1: Figure S1.** Kaplan-Meier survival curves showing risk of having a registered hospital visit due to either cardiovascular disease, type-2 diabetes, obesity, stroke, cancer, dementia, or depression; or the risk of combined morbidity (events from all examined diagnoses included in the analysis) by moderate-to-vigorous intensity physical activity (MVPA) tertiles.

**Additional file 2: Table S1.** Associations between physical activity and sedentary time and registered hospital visits due to combined cardiovascular disease (CVD), cancer and type 2-diabetes for participants without reported heart disease, cancer or diabetes at baseline.

## Data Availability

The datasets analyzed during the current study available from the corresponding author on reasonable request.

## Abbreviations

CI: Confidence interval
CVD: Cardiovascular disease
HR: Hazard ratio
IRR: Incidence rate ratio
MVPA: Moderate-to-vigorous physical activity
PA: Physical activity
SD: Standard deviation
