# Supplementary figures and images for: Accelerometry-assessed physical activity and sedentary time and associations with chronic disease and hospital visits - a prospective cohort study with 15 years follow-up
Source: Int J Behav Nutr Phys Act. 2019 Dec 9;16:125. doi: 10.1186/s12966-019-0878-2 (PMC6902520; doi:10.1186/s12966-019-0878-2)

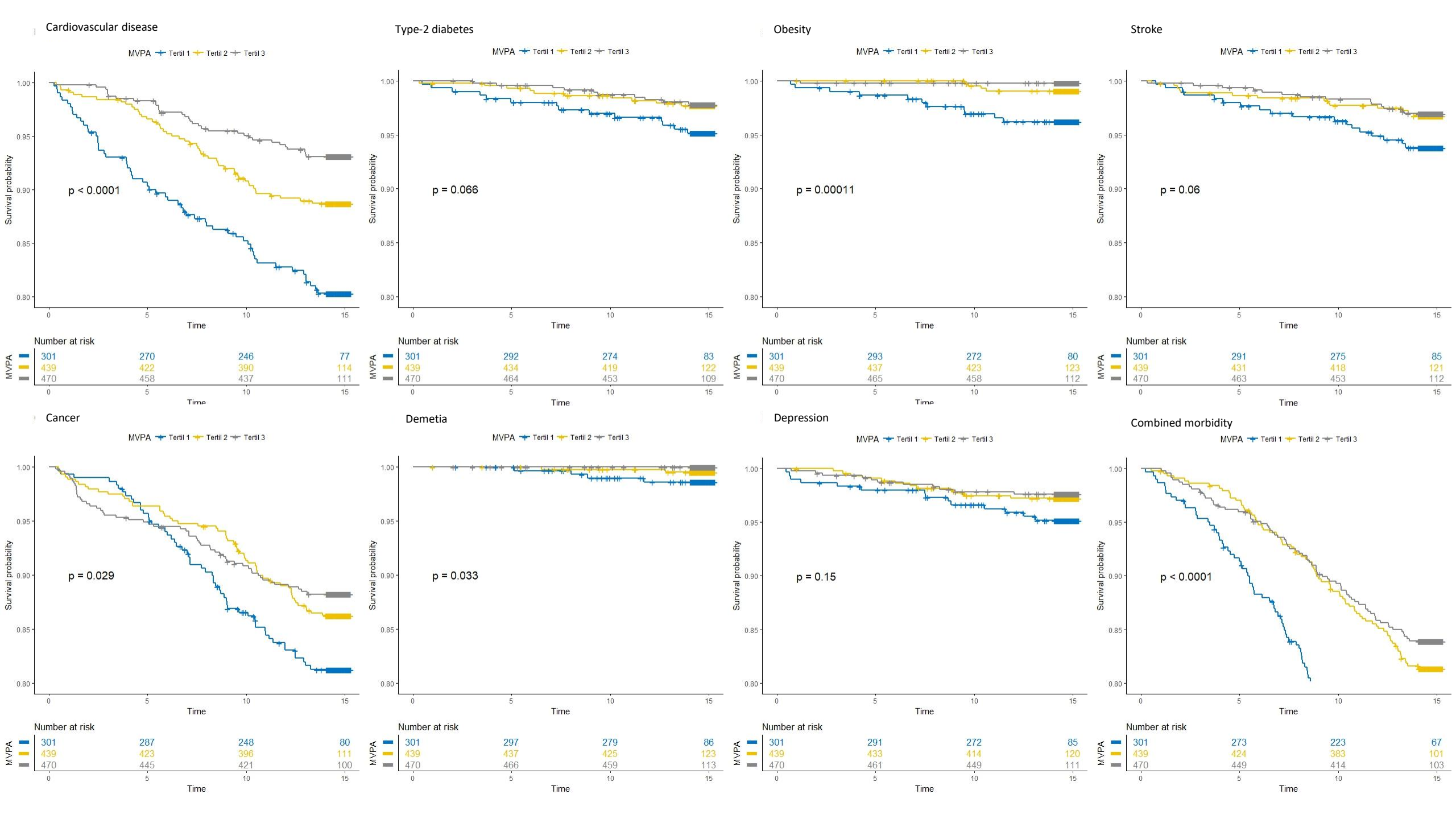

Supplement: Supplementary file 1 — Additional file 1: Figure S1. Kaplan-Meier survival curves showing risk of having a registered hospital visit due to either cardiovascular disease, type-2 diabetes, obesity, stroke, cancer, dementia, or depression; or the risk of combined morbidity (events from all examined diagnoses included in the analysis) by moderate-to-vigorous intensity physical activity (MVPA) tertiles. [file 12966_2019_878_MOESM1_ESM.pdf]
