# Supplementary material for: Accelerometry-assessed physical activity and sedentary time and associations with chronic disease and hospital visits - a prospective cohort study with 15 years follow-up
Source: Int J Behav Nutr Phys Act. 2019 Dec 9;16:125. doi: 10.1186/s12966-019-0878-2 (PMC6902520; doi:10.1186/s12966-019-0878-2)
Supplement: Supplementary file 2 — Additional file 2: Table S1. Associations between physical activity and sedentary time and registered hospital visits due to combined cardiovascular disease (CVD), cancer and type 2-diabetes for participants without reported heart disease, cancer or diabetes at baseline. [file 12966_2019_878_MOESM2_ESM.docx]

| **Table S1.** Associations between physical activity and sedentary time and registered hospital visits due to combined cardiovascular disease (CVD), cancer and type 2-diabetes for participants without reported heart disease, cancer or diabetes at baseline | | | | | | |
| --- | --- | --- | --- | --- | --- | --- |
| **CVD, cancer, diabetes** | **Model 1** | | **Model 2** | | **Model 3** | |
| n=1,132 / 247 events | HR | 95% CI | HR | 95% CI | HR | 95% CI |
|  | | | | | | |
| **Sedentary time** |  |  |  |  |  |  |
| Tertile 1 | 1 |  | 1 |  | 1 |  |
| Tertile 2 | **1.39** | **1.03, 1.88** | 1.03 | 0.76, 1.40 | 1.02 | 0.75, 1.39 |
| Tertile 3 | 1.25 | 0.90, 1.74 | 1.11 | 0.80, 1.56 | 1.10 | 0.79, 1.54 |
| **Light-intensity PA** |  |  |  |  |  |  |
| Tertile 1 | 1 |  | 1 |  | 1 |  |
| Tertile 2 | 1.08 | 0.79, 1.46 | 1.09 | 0.80, 1.48 | 1.09 | 0.80, 1.49 |
| Tertile 3 | 0.90 | 0.66, 1.22 | 0.98 | 0.71, 1.34 | 0.98 | 0.72, 1.35 |
| **Moderate-to-vigorous PA** |  |  |  |  |  |  |
| Tertile 1 | 1 |  | 1 |  | 1 |  |
| Tertile 2 | **0.66** | **0.49, 0.88** | 1.04 | 0.77, 1.44 | 1.06 | 0.77, 1.44 |
| Tertile 3 | **0.46** | **0.34, 0.64** | 0.76 | 0.55, 1.06 | 0.78 | 0.56, 1.09 |
| **Total activity counts** |  |  |  |  |  |  |
| Tertile 1 | 1 |  | 1 |  | 1 |  |
| Tertile 2 | 0.96 | 0.71, 1.31 | 1.07 | 0.79, 1.46 | 1.08 | 0.79, 1.47 |
| Tertile 3 | **0.60** | **0.44, 0.81** | 0.76 | 0.55, 1.04 | 0.77 | 0.56, 1.06 |
| Cox proportional-hazard models were used to estimate hazard ratios (HR) with 95% confidence intervals (CI). Model 1: crude. Model 2: adjusted for age, sex, smoking (missing=5), and education (missing=4). Model 3: adjusted for model 2 variables plus hypertension and arthritis (missing=13) at baseline. All models for sedentary time additionally adjusted for wear time. Statistically significant results shown in bold.  PA=physical activity | | | | | | |
